# European Wild Carnivores and Antibiotic Resistant Bacteria: A Review

**DOI:** 10.3390/antibiotics12121725

**Published:** 2023-12-13

**Authors:** Andreia Garcês, Isabel Pires

**Affiliations:** 1 Exotic and Wildlife Service from the Veterinary Hospital University of Trás-os-Montes and Alto Douro, Quinta dos Prados, 4500-801 Vila Real, Portugal; 2 Centre for Research and Technology of Agro-Environmental and Biological Sciences, CITAB, Inov4Agro, University of Trás-os-Montes and Alto Douro, UTAD, Quinta de Prados, 5000-801 Vila Real, Portugal; 3 Center of Animal and Veterinary Science CECAV University of Trás-os-Montes and Alto Douro, Quinta dos Prados, 4500-801 Vila Real, Portugal; ipires@utad.pt

**Keywords:** mammals, wild, carnivores, bacteria, antibiotics, contamination, resistance

## Abstract

Antibiotic resistance is a global concern that affects not only human health but also the health of wildlife and the environment. Wildlife can serve as reservoirs for antibiotic-resistant bacteria, and antibiotics in veterinary medicine and agriculture can contribute to the development of resistance in these populations. Several European carnivore species, such as wolves, foxes, otters, and bears, can be exposed to antibiotics by consuming contaminated food, water, or other resources in their habitats. These animals can also be indirectly exposed to antibiotics through interactions with domestic animals and human activities in their environment. Antibiotic resistance in wildlife can harm ecosystem health and also impact human health indirectly through various pathways, including zoonotic disease transmission. Moreover, the spread of resistant bacteria in wildlife can complicate conservation efforts, as it can threaten already endangered species. This review aims to describe the presence of antibiotic-resistant bacteria in wild carnivores in Europe.

## 1. Introduction

Antimicrobial resistance (AMR) is considered one of the leading public health problems of the 21st century [1]. Although AMR has always existed, the overuse and misuse of antibiotics have increased antibiotic-resistant strains [2]. In recent decades, selective pressure has been generated by the use of antibiotics in medicine, veterinary, and agricultural practices, which has been responsible for a significant increase in antibiotic resistance [3].

“One Health” is a concept wherein human, animal, and environmental health are interconnected [4]. One of the greatest problems with “One Health” is antimicrobial resistance. This problem affects these three groups simultaneously. Humans and domestic and wild animals can be hosts and spreaders of AMR bacteria. Moreover, bacteria are continuously exchanged between the different environmental niches [5,6].

Although most wildlife prefer to live far from humans, some species have adapted and can live in contact with domestic animals or humans in urban environments. Therefore, they can be recognized as potential indicators of AMR dissemination [7]. Wild animals usually do not receive antibiotics or veterinary care, except in cases of interventions in endangered animals, admissions to wildlife rehabilitation centers, or treatments during disease outbreaks [8]. Studies have shown that AMR in most wildlife is associated with environmental exposure to anthropogenic AMR contamination [8]. Air, water, land, and food are some of the sources of AMR [9]. Bodies of water, such as rivers, lakes, or seas, can be contaminated with industrial discharges, agricultural discharges (fecal sludge from farms), domestic sewage, discharges from hospitals (human and veterinary), and wastewater treatment plants, among others [8,10,11]. Fertilizers used in agriculture can be a source of AMR [8]. In addition to environmental pressures, there are intrinsic mechanisms in bacteria that may contribute to the development of antimicrobial resistance, such as bacterial permeability, efflux pumps, target receptor modification, or horizontal gene transfer between bacteria via mobile genetic elements (e.g., plasmids, transposons, integrons) [3,12]. The presence of AMR in wildlife is also associated with other factors, such as habitat use, foraging behavior, and species’ habitats [3,8]. Habitat destruction, the loss of biodiversity, climate change, the accumulation of toxic pollutants, and the invasion of exotic species and pathogens have also contributed to the spread of AMR [13].

Contact between anthropogenic source areas and wild animals has increased due to human expansion. Some animals—for example, foxes and hedgehogs—have adapted and now live and thrive in urban areas [1,14]. Animals in these areas can feed on human domestic waste [15]. These contacts can potentially contribute to the emergence of new pathogens and AMR in wildlife, which can promote higher mortality rates. When animals survive, they can become bacterial reservoirs and spread throughout the environment again [13,16]. A study performed in Botswana showed that the prevalence of AMR *Escherichia coli* was highest in carnivores (62.5%) and animals using urban habitats (25.6%) when compared to herbivores (9.1%) and animals using protected/rural habitats (9.0%) [8].

Despite the abundance of literature on AMR in the medical and veterinary fields, available studies focus mainly on some bacterial species, such as *Escherichia coli* or *Salmonella* spp., and some species of wild animals, mainly birds and mammals [6,7,8]. Carnivores are a very diverse group of species in Europe, with some populations living in remote areas and others in urban areas in close contact with humans [10,15].

This review aims to describe the presence of antibiotic-resistant bacteria in wild carnivores in Europe.

## 2. European Wild Carnivorous

Carnivora is an order of mammals that eats meat, by predation or necrophagy. They have specialized teeth for their meat-based diet, with fang-like canines, which they use to kill their prey and cut the meat into pieces [17,18]. Some animals in this order can also consume vegetation, insects (omnivores), and meat [17]. Carnivores can be found in diverse habitats, including cold polar regions, desert regions, forests, open seas, and urban areas [19]. The order Carnivora includes 16 families and 9 terrestrial families: Canidae, Felidae, Ursidae, Procyonidae, Mustelidae, Herpestidae, Viverridae, and Hyaenidae.

In Europe, there are approximately 63 species of carnivorous mammals, both terrestrial and marine. Some of these species are threatened according to the IUCN Red List of Threatened Species, such as the Iberian lynx (endangered) or the Balkan lynx (critically endangered) [17]. These include larger predators, such as wolves, bears, and lynxes, and smaller carnivores like foxes, weasels, and mustelids. Historically, throughout the continent, these species have all experienced a dramatic decline in their populations and distributions due to anthropogenic factors (hunting, habitat destruction, pollution) [18,20,21].

In Table 1, we present some information regarding the distribution, conservation status, and diet of some of the carnivorous species included in this review, to understand better the source of the acquisition of AMR strains of bacteria.

## 3. Antibiotic Resistance in Wild Carnivores

For this review, the inclusion criteria were as follows: studies only performed in free-range animals, species of European terrestrial carnivores, studies conducted in Europe, and studies that included bacteria, phenotypic resistance, and/or resistance genes.

**Figure 1:**
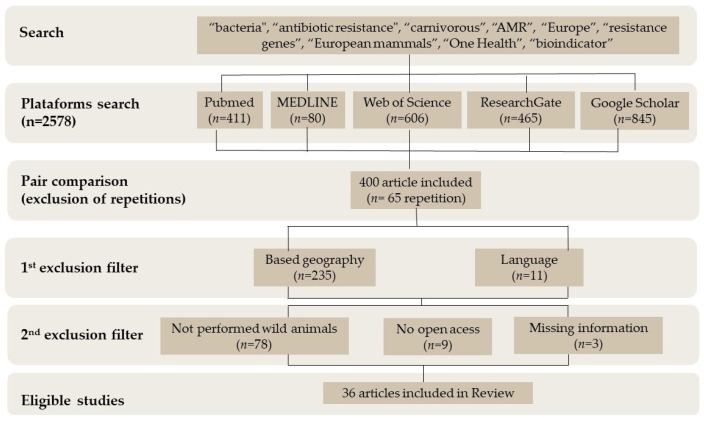
Flow diagram of data collection.

The initial search identified 2578 articles on the databases (ResearchGate, MEDLINE, PubMed, Web of Science, and Google Scholar) using the terms “bacteria”, “antibiotic resistance”, “carnivorous”, “AMR”, “Europe”, “resistance genes”, “European mammals”, “One Health”, and “bioindicator”. On the 2578 articles collected, a first screening was performed based on the information in the abstracts. A total of 2178 were excluded since they did not have the necessary information for the review. From the remaining 400, 65 were duplicates and therefore excluded. Another 235 were excluded due to geography (studies performed outside Europe). Eleven were removed due to language, since only English, Spanish, and Portuguese manuscripts were included in this review. With a secondary exclusion filter screening the full articles, 78 were excluded since they were not performed in wild animals or did not include all the information required, and 9 were not open-access full articles. Therefore, a total of 36 articles had all the information required and were included in this review (Figure 1). Table 2, Table 3, Table 4 and Table 5 is presented the information from the papers selected. 

### 3.1. Species and Spatial Distribution

The main families of carnivores where studies were carried out, in descending order, were as follows: 11.1% (*n* = 4) Ursidae, 16.6% (*n* = 6) Felidae, 19.4% (*n* = 7) Canidae, and 52.7% (n = 19) Mustilidae. The species with the most AMR studies was *Vulpes vulpes* with 12 studies, followed by *Lutra lutra* with 11 studies.

**Figure 2:**
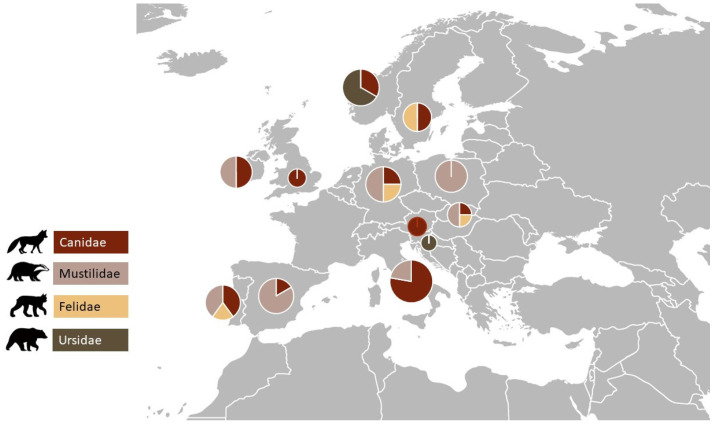
Distribution of the studies in the different European countries by carnivorous family group.

The countries where the studies were carried out, in ascending order, were as follows: 41.6% (*n* = 15) Portugal, 25% (*n* = 9) Italy, 8.3% (*n* = 3) Norway, 8.3% (*n* = 3) Germany, 8.3% (*n* = 3) Slovakia, 5.5% (*n* = 2) Ireland, 5.5% (*n* = 2) Slovenia, 5.5% (*n* = 2) Poland, 2.7% (*n* = 1) Austria, 2.7% (*n* = 1) Sweden, 2.7% (*n* = 1) United Kingdom. Figure 2 represents the number of studies by carnivorous species in each country included in this review.

### 3.2. Bacteria, Antibiotic Resistance Pattern, and Resistance Genes

**Figure 3:**
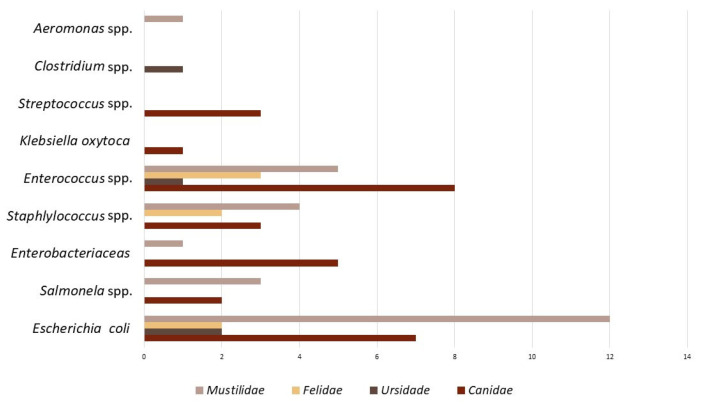
Bacteria species that predominate in the 36 studies in antibiotic resistance in wild carnivores.

Most of the studies were performed in fecal samples or rectal swabs; therefore, the bacteria isolated mostly were microbiota from the gut microflora (Figure 3).

**Figure 4:**
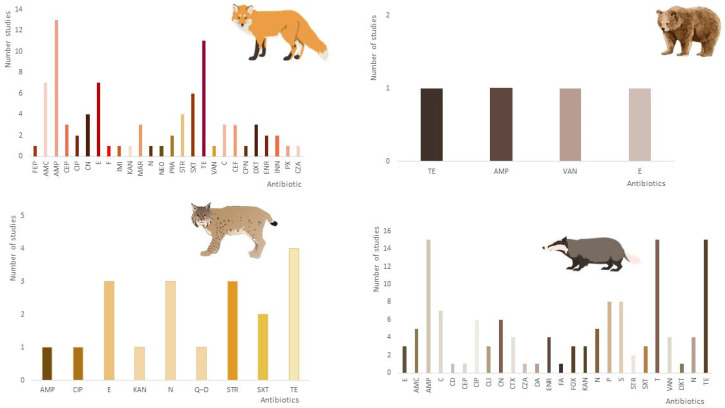
Occurrence of phenotypic antimicrobial resistance profile of bacteria in wild carnivores based on the articles included in this review (AMC: amoxicillin/clavulanic acid; AMP: ampicillin; STR: streptomycin; E: erythromycin; ENR: enrofloxacin; E: erythromycin; BE: benzylpenicillin; C: chloramphenicol; CD: clindamycin; CEF: ceftiofur; CEP: cephalothin; CN: gentamicin; CPN: cephalexin; CRO: ceftriaxone; CTX: cefotaxime; DXT: doxycycline; F: nitrofurantoin; IMI: imipenem; INN: cefovecin; KF: cephalothin; MAR: marbofloxacin; NEO: neomycin; PRA: pradofloxacin; PX: cefpodoxime; SXT: trimethoprim/sulfamethoxazole; TE: tetracycline; N: nalidixic acid; CIP: ciprofloxacin; KAN: kanamycin; VAN: vancomycin; Q–D: quinupristin–dalfopristin; CZA: ceftazidime; FEP: cefepime; FOX: cefoxitin: FA: fusidic acid; P: penicillin; T: tobramycin).

Regarding phenotype resistance, Figure 4 considers the number of articles that describe, in particular, each type of antibiotic resistance reported in the various Carnivora families: Canidae, Ursidae, Felidae, and Mustelidae. The studies are also summarized in Table 2 and Table 3. Many papers report multi-resistant bacteria. The methodology used in these articles was very similar, using the disk diffusion method (DDM) as antibiotic sensitivity testing. All the terminology was also standardized to be included in this graphic.

Based on the information collected in the different articles regarding the antibiotic resistance phenotype, it was possible to observe that three of the carnivore families—Canidae, Felidae, and Mustilidae—presented high levels of resistance to ampicillin and tetracyclines (Figure 4). In the case of the Ursidae family, it is impossible to extract any valid information due to the limited number of studies, and the resistance pattern is quite similar.

## 4. Carnivores and Antibiotic Resistance

Antibiotic-resistant bacteria can be acquired by carnivorous species in several ways, mainly through direct and indirect exposure to these resistant strains from anthropogenic sources and domestic animals [10,62]. Normally, these animals are not treated with antibiotic therapy, except in some particular cases in which some individuals are admitted to wild animal rehabilitation centers due to illness or trauma. However, even in these cases, exposure to these agents is brief [64,65]. Major predators can generally travel great distances across the territory for food. They can disperse AMR over large areas, a key element of AMR dynamics in the ecosystem [66].

Some species of animals are natural carriers of AMR bacteria. For example, European hedgehogs (*Erinaceus europaeus*) are natural carriers of MRSA that have been selected as a response to the presence of b-lactam-producing microorganisms (*Trichophyton erinacei*) in the microbiome of this animal [67].

The greatest problem is the contamination of the environment with antibiotic resistance determinants and resistance drivers (e.g., antibiotic residues, pesticides, heavy metals) from agriculture, waste disposal, or the disposal of wastewater of human and veterinary origin [8]. The dispersion of these agents in the environment is a public health problem, as it can lead to the emergence and proliferation of pathogens that are difficult or impossible to treat [8,63]. The dispersion of these agents has negative economic and health consequences for humans and animals [68].

Several studies have already been carried out on the presence and impact of antibiotic-resistant bacteria in wildlife across various vertebrates, from birds to reptiles [69,70]. Based on already available data, the prevalence of antibiotic-resistant bacteria depends on multiple factors, such as habitat use, the foraging strategy of the species, behavior, and territory [8].

Unfortunately, not all European carnivore species are represented in this review, as no data are available for some of them [62]. This article’s main limitation is that it does not allow a realistic comparison between different species. This demonstrates that it is necessary to collect more data on other species and in different regions, in the long term, to compare the impact that the use and abuse of antibiotics have on these animals.

Most of the studies included in this review were conducted in Southern and Eastern European countries (Figure 3). This fact may be partly associated with the greater diversity of animals in these regions. However, it is also possible that it is related to the fact that several Southern and Eastern European countries have reported higher levels of antibiotic resistance in livestock, often linked to differences in agricultural practices, regulations, and intensive livestock production. In addition, many of these regions are highly industrialized [71,72].

Concerning the available data, it is possible to observe that most studies were conducted on small mammals (Figure 3), mainly from the Mustilidae family. This may be associated with the fact that large carnivore populations (wolf, bear, wolverine, lynx) have declined in Europe and their numbers are very small [62,73]. Most of these populations are threatened and protected by law [34,52]; therefore, it is necessary to access samples from these individuals to carry out studies [37,74]. In addition to being threatened, some species, such as polar bears, live in very remote areas, difficult to access and with harsh climates [25,50].

One of the most represented species is the red fox (*Vulpes vulpes*). This may be associated with its omnivorous diet and adaptability to urban centers. Currently, these animals can be easily found in several European cities, feeding on human waste and in close contact with domestic animals [26,42]. Due to this behavior, they can be excellent bioindicators of the presence of AMR in the environment [47,75]. Animals such as foxes, which live close to humans and often depend on their waste for food, are more susceptible to these agents. Similar studies in birds in Southern France detected carbapenem-resistant *E. coli* isolates in yellow-legged gulls (*Larus michahellis*) feeding in landfills. However, no isolates were obtained from slender-billed gulls (*Chroicocephalus genie*) provided from deep-sea fish [76]. Another source of contamination may be the prey of small species, such as rodents or insects, which may represent a link between humans/domestic animals and predators [76]. In the case of flies, these are usually found in contaminated waste and can travel quite a long distance as vectors of AMR bacteria, infecting wild/domestic animals and humans [66]. Moreover, scavenging contaminated carcasses or consuming peridomestic prey may promote exposure to AMR [8].

The most observed species of bacteria were *E. coli* and *Enterococcus* spp. (Figure 3) in general in the four families of carnivores. *E. coli* prevailed in all families except Felidae, where *Enterococcus* spp. was the most prominent bacterial species. The results were expected since most samples originated from feces or rectal swabs [52,72].

Although the use of antibiotics in livestock farming has been reduced to minimal use under EU regulations [1], sulfamethoxazole, ampicillin, and tetracycline were the primary resistance types reported in livestock animals [77,78]. Tetracycline and ampicillin are also some of the most commonly used antibiotics in human medicine, and resistance to these isolates is frequently reported [79]. In the data collection, almost all carnivore families have resistance to ampicillin, tetracyclines, and sulfonamides. This may be an indication that livestock and humans may be the potential sources of these forms of AMR in wild carnivores.

Some resistant bacteria are more dangerous than others, as in the case of extended-spectrum beta-lactamase (ESBL)-producing bacteria, vancomycin-resistant Enterococci (VRE), and methicillin-resistant *Staphylococcus aureus* (MRSA) [44,62]. Infections with these bacteria are challenging to treat and can lead to severe complications [62,63]. The presence of ESBL has been reported in Iberian wolves [32,34], red foxes, badgers [58], and European otters [54,56]; VRE in Iberian wolves [36]; and MRSA in European otters and the European lynx [45,57]

The mecA gene is the main genetic determinant responsible for methicillin resistance in *Staphylococcus aureus.* In the studies presented where MRSA was observed, it was isolated from *mecC*, which is homologous for *mecA* [45,57]. Vancomycin resistance in Enterococcus is commonly associated with two genes, *vanA* and *vanB*. These genes were isolated in the Iberian wolf [37]. Several genes are associated with ESBL production, such as *TEM*, *SHV*, and *CTX-M.* These genes have been identified in Iberian wolves [32,34], red foxes, badgers [58], and European otters [54,56]. Other important genes are *bla_CTX-M_*, *bla_CMY_*, *tetM*, and *ermB*, also isolated in several species, indicating that bacteria or resistance originated in human or domestic animals [78,79].

The correlation between AMR and the United Nations Sustainable Development Goals (SDGs) is a global health concern and has specific implications for wildlife populations, including carnivores in Europe [80]. As these animals play vital roles in ecosystems, their health is interconnected with the broader environmental and human health goals outlined in the SDGs. In the context of carnivores, AMR can have cascading effects on ecosystems. For example, the use of antibiotics in domestic animals, which is linked to AMR, can indirectly impact carnivores through food chain dynamics [81]. Additionally, the spread of antibiotic-resistant bacteria in the environment, including water bodies, can affect carnivores that rely on these resources. This aligns with SDG 15 (Life on Land) and SDG 14 (Life Below Water), emphasizing the importance of safeguarding terrestrial and aquatic ecosystems. Moreover, the potential transmission of antibiotic-resistant bacteria between wildlife, livestock, and humans underscores the interconnectedness of SDG 3 (Good Health and Well-Being). Efforts to mitigate AMR in carnivores involve understanding and addressing the factors contributing to the spread of resistance, emphasizing the need for interdisciplinary approaches that span the environmental, veterinary, and human health domains. In the broader context of SDG 17 (Partnerships for the Goals), collaboration between environmental scientists, veterinarians, public health experts, and policymakers becomes crucial [80]. Shared knowledge and coordinated efforts are necessary to develop strategies that protect carnivores, ecosystems, and public health from the threats posed by AMR [81]. Addressing AMR in carnivores aligns with the holistic and interconnected approach of the SDGs, recognizing that the health of wildlife is intrinsically linked to broader sustainability and well-being goals for the planet and its inhabitants [82].

## 5. Conclusions

Studies have shown that AMR can be found in various wildlife populations, including wild mammal carnivores, most likely of anthropogenic origin. This poses a risk to these animals, humans, and other animals that come into contact with them. While predicting the exact future of AMR in wild carnivores is challenging, the issue will likely continue to be a concern if proper measures are not taken to address it. Several studies have identified AMR bacteria and antibiotic resistance genes in wild carnivores, including foxes, raccoons, and wild felids, as it was possible to conclude with this review. Antibiotic resistance in these animals is often linked to anthropogenic activities, environmental contamination, and interactions with human-influenced areas.

The future of AMR in wild carnivores depends on understanding the extent and impact of antibiotic resistance in these populations, which is essential for the development of effective strategies to mitigate its spread. Monitoring the prevalence of antibiotic-resistant bacteria in these populations, studying the mechanisms of resistance, and identifying the sources of antibiotic exposure are crucial measures in addressing this issue. Wild carnivores can be useful bioindicators of AMR in the environment. Moreover, promoting the responsible use of antibiotics in veterinary medicine and agriculture, and implementing measures to reduce environmental contamination with antibiotics, can help to minimize the emergence and spread of antibiotic resistance in wild mammal carnivores and other wildlife populations. Collaboration between wildlife conservationists, veterinarians, and public health experts is essential to develop comprehensive strategies to preserve both animals, especially threatened species, and human health in the face of antibiotic resistance under the “One Health” concept.

## Figures and Tables

**Table 1:** Species, family, distribution, diet, habitat, behavior, and conservation status (LC—Least Concern, V—Vulnerable, NT—Near Threat) of wild carnivore species from Europe.

Species	Family	Distribution	Diet	Habitat	Behavior	Conservation Status	Ref.
Beech marten (*Martes foina*, *Erxleben*, 1777)	*Mustelidae*	Europe, except most Mediterranean islands, the Balkan peninsula, the Scandinavian peninsula, and the United Kingdom	Plants, fruit, rats, mice, small mammals, birds	Urban areas, forest habitats, and rural areas	Crepuscular and nocturnal	LC	[22]
European polecat (*Mustela putorius*, Linnaeus, 1758)	*Mustelidae*	Western European Russia, Western Belarus, Western Ukraine, Central and Western Europe, and North Africa	Lagomorphs, small rodents, amphibians, birds, reptiles, and insects	Riparian and agricultural areas to meadows and forest areas	Nocturnal	LC	[23]
Brown bear (*Ursus arctos*, Linnaeus, 1758)	*Ursidae*	Europe, Asia, Atlas Mountains, North America	Omnivore	Mountain woodlands, forest	Crepuscular	LC	[24]
Polar bear (*Ursus maritimus*, Phipps, 1774)	*Ursidae*	Greenland, Canada, Alaska, Russia, and the Svalbard Archipelago of Norway	Seals, walruses, sea birds, eggs, small mammals, fish, reindeer/caribou, seaweed/kelp, land plants	Ice fields	Diurnal	V	[25]
Red fox (*Vulpes vulpes*, Linnaeus, 1758)	*Canidae*	Northern hemisphere	Plants, rodents, birds, leporids, porcupines, raccoons, opossums, reptiles, insects, invertebrates	Scrubland, forest, agricultural fields, urban areas	Nocturnal	LC	[26]
European badger (*Meles meles*, Linnaeus, 1758)	*Mustelidae*	Europe (except Scandinavia), Russia, and parts of Asia	Omnivores: plants, earthworms, large insects, small mammals, fruits	Deciduous, mixed, and coniferous forests, agro-silver-pastoral landscapes, Mediterranean scrub forests, and open areas with patches of riparian vegetation	Crepuscular and nocturnal	LC	[27]
European otter (*Lutra lutra*, Linnaeus, 1758)	*Mustelidae*	Eurasia, North Africa, the Middle East, Sri Lanka, a part of India, and Indonesia	Fish, amphibians, insects	Rivers, streams, marshes, lagoons, and reservoirs	Nocturnal	NT	[28]
Apennine wolf (*Canis lupus italicus*, Altobello, 1921)	*Canidae*	Italy, France, Spain, Switzerland	Roe deer, wild boar, red deer, livestock sheep, horses, Mouflon, Italian hare, birds, invertebrates, fruit, berries, grasses, herbs, and garbage	Temperate coniferous forests	Crepuscular, diurnal	V	[29]
Iberian wolves (*Canis lupus signatus*, Cabrera, 1907)	*Canidae*	Portugal, Spain	Wild boars, rabbits, roe deer, red deer, ibexes, small carnivores, and fish	Temperate forests	Crepuscular, diurnal	EN	[30]
Iberian Lynx (*Lynx pardinus*, Temminck, 1827)	*Felidae*	Portugal, Spain	Rabbits, small rodents	Mediterranean forests of woodland and shrubland interspersed with natural and artificial pastures	Crepuscular and nocturnal	EN	[31]
Golden jackal (*Canis aureus*, Linnaeus, 1758)	*Canidae*	Southeastern Europe, Moldova, Asia Minor, and the Caucasus	Omnivorous diet, plants, fruit, rodents, rabbits	Valleys, beside rivers canals, lakes, seashores	Crepuscular	LC	[32]

**Table 2:** Antibiotic resistance in animals from the family Canidae regarding species, country, year, type of sample, bacteria isolated, antibiotic resistance, and resistance genes.

Species	Country	Year	Type of Sample	Isolated Bacteria	Antibiotic Resistance *	Resistance Genes	Ref.
Apennine wolf (*Canis lupus italicus*)	Italy	2015–2017	Feces	*Citrobacter* spp., *Escherichia coli*, *Hafnia alvei*, *Salmonella* spp., *Serratia* spp.	AMC, AMP, STR	*n/a*	[33]
	Italy	2017	Feces	*n/a*	TE	*tetA*, *tetP*	[7]
	Italy	2022	Endocardial swab, lung, thoracic effusion	*Staphylococcus pseudointermedius*, *Enterococcus faecalis*, *E. coli*	AMC, E, ENR, MAR, CXT, C, SXT, TE, P, DXT	*n/a*	[34]
	Italy	2022	Peritoneal effusion, lung, endocardial swab, liver parenchyma, pleural effusion	*Klebsiella oxytoca*	AMP	*n/a*	[34]
	Italy	2022	Forearm wound, exposed fracture	*Streptococcus dysgalactiae* spp. *equisimilis*, *Leclercia adecarboxilara*	AMP, C, CEF, CEP, CN, CPN, DX, ENR, INN, MAR, PRA, PX, SXT, TE	*n/a*	[34]
	Italy	2022	Carpal wound, intraarticular swab	*Streptococcus canis*, *E. coli*, *Pseudomonas aeruginosa*	AMP, C, CPN, CEP, DXT, ENR, INN, MAR, NEO, PRA, SXT, TE, AMC, CEF, CN, CPN, IMI, F	*n/a*	[34]
Iberian wolves (*Canis lupus signatus*)	Portugal	2008–2010	Feces	*E. coli*	TE, AMP, STR, CEP, N, SXT, CIP	*cdt*, *chuA*, *cvaC*, *eaeA*, *paa*, *bfpA*, *bla_CTX-M-1_*, *bla_CTX-M-9_*	[35]
	Portugal	2008–2009	Feces	*Enterococcus faecium*, *E. hirae*, *E. faecalis*, *E. durans*	AMP, TE, STR	*tetM*, *tetL*, *ermB*, *bla_TEM_*, *tetA*, *tetB*, *aadA*, *strA-strB*	[36]
	Portugal	2008–2010	Feces	*E. faecium*, *E. gallinarum*	TET, VAN, AMP, E, KAN	*vanC1*, *vanA*, *tetM*, *ermB; aph(3′)-IIIa*, *tet(L); Tn916*, *hyl*	[37]
Golden jackal (*Canis aureus*)	Italy	2023	Lung, liver, spleen, kidney, and intestine	*n/a*	*n/a*	*tetM*, *tetP*, *mcr-1*, *tetA*, *tetL*, *tetM*, *tetO*, *sul3*, *bla_TEM−1_*	[38]
Red Fox (*Vulpes vulpes*)	Portugal	2008–2009	Feces	*E. coli*	STR, TE, SXT, AMP	adA, tetA, tetB, sul1, bla_TEM_	[39]
	Portugal	2008–2009	Feces	*E. faecium*	TE	tetM, tetL, ermB, aph(30)-IIIa	[39]
	Portugal	2008–2009	Feces	*E. faecium*, *E. durans*	TE, E	*ermB*, *tetM*, *tetL*, *Tn916*	[40]
	Ireland	2018–2019	Fecal, nasopharyngeal swabs	*E. coli*	CZA, TE, SXT, CIP, AMP, FEP	*n/a*	[41]
	Norway	2006	Fecal	*E. coli*	SXT, TE, CIP, N	*n/a*	[1]
	Portugal	2017–2019	Fecal	*E. coli*, *Enterococcus* spp.	TE, C, CD, CN, AMC, AMP, BE, CEF, CEP, CZA, CPN, CRO	*bla_TEM_*, *ermB*, *aadA*, *tetM*, *tetW*, *tetL*, *drfA1*, *drfA17*	[42]
	Italy	2016–2018	Fecal	*E. coli*	*n/a*	*eaeA*, *hlyA*, *stx1*, *and stx2,*	[43]
	Italy	2002–2010	Rectal swab	*Salmonella enterica*, *S. typhimurium*	AMC, TE, AMP, ENR	*n/a*	[44]
	Germany, Austria, Sweden	2013, 2006, 2005, 2014	Nasal swab	*S. aureus*	*n/a*	*gapA*, *katA*, *CoA*, *Spa*, *sbi*, *nuc1*, *sarA*, *saeS*, *vraS*, *agrl*, *hid*	[45]
	Slovakia	2020	Feces	*Enterococcus* spp.	TE, AMP, VAN, E	*n/a*	[46]
	Spain	2012–2015	Nasal and rectal swabs	*Staphylococcus* spp.	CD, F, AMP, BE, FOX, FA, NEO	*n/a*	[47]
	Italy	2017–2019	Oral, skin, rectal, tracheal swab, feces	*K. oxytoca*	AMP, CD	*n/a*	[48]
	UK	2007–2008	Tissues	*S. sciuri* group, *S. equorum*, *S. capitis*	MET, CL, AMC, AMP, ENR, FD, DA, TET	*mecA*	[8]

* AMC: amoxicillin/clavulanic acid; AMP: ampicillin; STR: streptomycin; E: erythromycin; ENR: enrofloxacin; E: erythromycin; BE: benzylpenicillin; C: chloramphenicol; CD: clindamycin; CEF: ceftiofur; CEP: cephalothin; CN: gentamicin; CPN: cephalexin; CRO: ceftriaxone; CTX: cefotaxime; DXT: doxycycline; F: nitrofurantoin; IMI: imipenem; INN: cefovecin; KF: cephalothin; MAR: marbofloxacin; NEO: neomycin; PRA: pradofloxacin; PX: cefpodoxime; SXT: trimethoprim/sulfamethoxazole; TE: tetracycline; N: nalidixic acid; CIP: Ciprofloxacin; KAN: kanamycin; VAN: vancomycin; Q–D: quinupristin–dalfopristin; CZA: ceftazidime; FEP: cefepime; FOX: cefoxitin: FA: fusidic acid; DA: clindamycin; MET: methicillin.

**Table 3:** Antibiotic resistance in animals from the family Ursidae regarding species, country, year, type of sample, bacteria isolated, antibiotic resistance, and resistance genes.

Species	Country	Year	Type of Sample	Isolated Bacteria	Antibiotic Resistance *	Resistance Genes	Ref.
Polar bear (*Ursus maritimus*)	Svalbard	2014	Fecal	*Clostridiales*	*n/a*	*bla_TEM_*	[49]
	Svalbard	2004–2006	Fecal	*Clostridiales*, *Firmicutes*, *E. coli*	*n/a*	*bla_TEM_*	[50]
Brown bears (*Ursus arctos*)	Slovenia	2010–2012	Fecal	*E. coli*	*n/a*	*fimH*, *ompT*, *kpsMT*, *ibeA*, *traT*	[51]
	Slovakia	2020	Feces	*Enterococcus* spp.	TE, AMP, VAN, E		[46]

* AMP: ampicillin; E: erythromycin; TE: tetracycline; VAN: vancomycin.

**Table 4:** Antibiotic resistance in animals from the family Felidae regarding species, country, year, type of sample, bacteria isolated, antibiotic resistance, and resistance genes.

Species	Country	Year	Type of Sample	Isolated Bacteria	Antibiotic Resistance *	Resistance Genes	Ref.
Iberian Lynx (*Lynx pardinus*)	Portugal	2008–2010	Feces	*E. casseliflavus*	TE, Q–D, E, STR	*vanC2*, *tetM*, *ermB*, *hyl*, *cylA*, *cylL,*	[37]
	Portugal	2008–2010	Feces	*Enterococcus* spp., *E. coli*	TE, E, STR, N, SXT,	*cpd*, *cylB*, *and cylL*, *bla_TEM_*, *tetA*, *aadA*, *cmlA*, *dfrA1 + aadA1*, *dfrA12 + aadA2*, *fimA*	[36]
	Portugal	2008–2010	Feces	*Enterococcus* spp.	TE, E, KAN, N	*tetM*, *tetL*, *ermB*, *aac (6′)-Ie-aph(2″)-Ia*, *ant(6)-Ia*, *aph(3′)-IIIa*	[52]
	Portugal	2008–2010	Feces	*E. coli*	TE, STR, SXT, N, AMP, CIP	*bla_TEM_*, *bla_SHV_*, *tetA*, *tetB*, *aadA*, *strA-strB*, *aac(3)-II*, *aac (3)-IV*, *aadA1*, *dfrA1 + aadA1*, *estX + psp + aadA2*, *aer*, *cnf1*, *fimA*, *papC*, *papG-allele III*	[52]
Wild cat (*Felis silvestris*)	Germany	2014	Nasal swab	*S. aureus*	*n/a*	*gapA*, *katA*, *CoA*, *Spa*, *sbi*, *nuc1*, *sarA*, *saeS*, *vraS*, *agrl*, *hid*	[45]
Lynx (*Lynx lynx*)	Sweden	2006	Liver tissue	*S. aureus*	*n/a*	*gapA*, *katA*, *CoA*, *Spa*, *sbi*, *nuc1*, *sarA*, *saeS*, *vraS*, *agrl*, *hid*, *agrlV*, *mecC*	[45]

* AMP: ampicillin; STR: streptomycin; E: erythromycin; SXT: trimethoprim/sulfamethoxazole; TE: tetracycline; N: nalidixic acid; CIP: ciprofloxacin; KAN: kanamycin; Q–D: quinupristin–dalfopristin.

**Table 5:** Antibiotic resistance in animals from the family Mustelidae regarding species, country, year, type of sample, bacteria isolated, antibiotic resistance, and resistance genes.

Species	Country	Year	Type of Sample	Isolated Bacteria	Antibiotic Resistance *	Resistance Genes	Ref.
Eurasian otter (*Lutra lutra*)	Portugal	2006–2008	Feces	*E. faecalis*, *E. faecium*, *E. durans*	*n/a*	*ace*, *acm*, *ebpABC*, *gelE*, *cylA*, *tetM*, *pbp5*, *vanB*, *vanD*, *aac(60)-Ie-aph*	[53]
	Portugal	2015–2016	Feces	*Enterococcus* spp.	AMC, AMP, C, CN, DA, ENR, P, TE, VAN	*n/a*	[54]
	Portugal	2006	Feces	*Aeromonas hydrophila*, *A. hydrophila/caviae*, *A. sobria*	P, CLI, E, VAN, AMP	*n/a*	[55]
	Portugal	2006–2008	Feces	*S. arizona*, *S. pullorum*, *S. choleraesuis arizona*	AMC, C, P, AMP, CL, ENR, GN, NA, S, TE	*n/a*	[16]
	Portugal	2009	Feces	*E. coli*, *Enterococcus* spp.	CTX, ENR, S	*n/a*	[13]
	Spain	2018–2021	Feces	*E. coli*, *Pseudomonas fluorescens*, *Hafnia alvei*, *Serratia marcescens*	CIP, ENR, CN, SXT, TE, C	*ermB*, *bla_CTX-M-15_*, *tetM*, *bla_CMY-2_*, *tetM*	[56]
	Germany	2000–2012	Nasal and perineal swabs	*S. aureus*	AMC, AMP, P,	*mecC*	[57]
	Portugal	2018–2019	Feces	*E. coli,*	AMP, SXT, TE, CTX, KAN, CN, PX, DXT, T	*aac(3)-IV*, *aph(4)-Ia*, *aph(6)-Id*, *bla_TEM-1B_*, *lnu(F)*, *tet(B)*, *aac(3)-Iva*, *aadA1*, *aac(2′)-Iia*, *qnrB19*, *adA5*, *aph(3″)-Ib*, *catA1*, *qnrB19*, *qnrB82*, *sulII*, *dfrA17*	[58]
	Slovakia	2020	Feces	*Enterococcus* spp.	TE, E, AMP, VAN	*n/a*	[46]
	Spain	2012–2015	Nasal and rectal swabs	*Staphylococcus* spp.	N, P, FOX, FA	*n/a*	[49]
	Spain	2015–2015	Fecal	*E. coli*	AMP, TET, SXT	*dfrA1 aadA1 qacE 1*, *sul1*, *sul2*, *tetA*	[59]
Badger (*Meles meles*)	Ireland	2018–2019	Fecal, nasopharyngeal swabs	*Salmonella* spp., *E. coli*	AMP, CZA, CEP, CTX	*n/a*	[60]
	Spain	2016–2017	Swabs	*E. coli*	CIP, N, C, S, T	*bla_SHV-12_*	[61]
	Poland	2014–2018	Rectal swabs	*E. coli*	AMP, S, KAN, C, CIP, S, N, TE	*aph(3¢)-Ia*, *strA*, *aph(3¢)-Ia*, *sul2*, *tetA*, *tetB*, *floR*, *cat*, *sul3*	[62]
	Germany	2011	Pharyngeal swab	*S. aureus*	*n/a*	*gapA*, *katA*, *CoA*, *Spa*, *sbi*, *nuc1*, *sarA*, *saeS*, *vraS*, *agrl*, *hid*	[45]
	Spain	2015–2015	Fecal	*E. coli*	AMP, TE	*tetB*	[59]
	Spain	2012–2015	Nasal and rectal swabs	*Staphylococcus* spp.	N, P, FOX, FA, CLI	*n/a*	[63]
Beech marten (*Martes foina*)	Poland	2014–2018	Rectal swabs	*E. coli*	AMP, STR, KAN, C, CN, CIP, S, N, TE, CTX	*strA*, *sul1*, *sul2*, *tetA*, *tetB*, *aph(3¢)-Ia*, *floR*, *cat*, *bla_TEM-135_*	[62]
	Spain	2012–2015	Nasal and rectal swabs	*Staphylococcus* spp.	N, PEN, FOX, TE	*n/a*	[49]
	Spain	2015–2015	Fecal	*E. coli*	AMP, NAL, CIP	*bla_TEM-1b_*	[59]
	Spain	2016–2017	Swabs	*Citrobacter freundii*	CIP, NAL, GEN, TET, SUL, TMP	*black my_-2_*, *bla_SHV-12_*	[61]
European pine marten (*Martes martes*)	Slovakia	2020	Feces	*Enterococcus* spp.	TE, E, AMP, VAN	*n/a*	[46]
	Italy	2002–2010	Rectal swab	*Salmonella* spp.	AM, AMC, TE	*n/a*	[44]
	Italy	2017–2019	Oral, skin, rectal, tracheal swab, feces	*E. coli*	AMP, CD	*n/a*	[48]
European polecat (*Mustela putorius*)	Poland	2014–2018	Rectal swabs	*E. coli*	AMP, STR, S, TET	*strA*, *sul2*, *tetA*	[62]

* AMC: amoxicillin/clavulanic acid; AMP: ampicillin; STR: streptomycin; E: erythromycin; ENR: enrofloxacin; E: erythromycin; BE: benzylpenicillin; C: chloramphenicol; CD: clindamycin; CEF: ceftiofur; CEP: cephalothin; CN: gentamicin; CPN: cephalexin; CRO: ceftriaxone; CTX: cefotaxime; DXT: doxycycline; F: nitrofurantoin; IMI: imipenem; INN: cefovecin; KF: cephalothin; MAR: marbofloxacin; NEO: neomycin; PRA: pradofloxacin; PX: cefpodoxime; SXT: trimethoprim/sulfamethoxazole; TE: tetracycline; N: nalidixic acid; CIP: ciprofloxacin; KAN: kanamycin; VAN: vancomycin; Q–D: quinupristin–dalfopristin; CZA: ceftazidime; FEP: cefepime; FOX: cefoxitin: FA: fusidic acid; P: penicillin; T: tobramycin.
